# Involvement of an alternatively spliced mitochondrial oxodicarboxylate carrier in adipogenesis in 3T3-L1 cells

**DOI:** 10.1186/1423-0127-16-92

**Published:** 2009-10-13

**Authors:** Masashi Niimi, Lian Tao, Shi-Hua Lin, Jun Yin, Xiaoyun Wu, Hiroyuki Fukui, Junichi Kambayashi, Jianping Ye, Bing Sun

**Affiliations:** 1 Otsuka Maryland Medicinal Laboratories, Inc, Rockville, Maryland, USA; 2 Department of Molecular Pharmacology, Graduate School of Health Biosciences, University of Tokushima, Japan; 3 Pennington Biomedical Research Center, Louisiana State University, Baton Rouge, Louisiana, USA; 4 Department of Medicine, University of Alabama at Birmingham School of Medicine, Birmingham, Alabama, USA

## Abstract

**Background:**

Adipogenesis is a complex process that involves many genes/proteins at different stages of differentiation. In order to identify genes critical for adipogeneis, we took a novel approach based on phenotype change of individual cell, to search for genes with regulatory roles in adipogenesis genome-wide in 3T3-L1 cells.

**Methods:**

Lentivirus-based inducible random homologous knockdown was used for the screening of functional gene that altered lipid formation in the adipocyte during differentiation.

**Results:**

In the present study, we reported the identification of an alternatively spliced mitochondrial oxodicarboxylate carrier (ODC), so named ODC-AS. ODC-AS is different from ODC by replacing 22 amino acids with 29 amino acids at the N-terminal. ODC was widely expressed in most tissues in mouse as determined by multi-tissue cDNA panel polymerase chain reaction. However, ODC-AS was only detected in adipose tissue and in iris and sclera-choroid complex of the eye. The expression of ODC-AS in 3T3-L1 was detected after the induction of differentiation, and reached a peak at day 4 and then reduced thereafter, whereas no ODC transcript detected in the cells neither before nor after differentiation. Knocking down of ODC-AS expression by RNA interference led to significant reduction in lipid accumulation as determined by triglyceride measurement and Nile Red staining, as well as adipogenic marker CEBPα, PPARγ, aP2 and CD36. Although both ODC and ODC-AS are expressed in white and brown adipose tissues, only the expression of ODC-AS was down-regulated in brown adipose tissue by cold exposure.

**Conclusion:**

These results implicate that ODC-AS may promote lipid accumulation during adipocyte differentiation and play an important role in the regulation of lipid metabolism in adipose tissues.

## Background

Obesity is a serious health hazard and is a risk factor for many kinds of metabolic abnormalities such as dyslipidemias, diabetes, insulin resistance and cardiovascular diseases. Obesity is essentially due to the excessive accumulation of triglyceride (lipogenesis) in adipocytes as well as elevated adipogenesis resulting in the formation of new adipocytes from precursor cells. Identification of the genes important for adipogenesis and lipogenesis would potentially lead to intervention to prevent or treat obesity.

Differentiation of 3T3-L1 preadipocytes into adipocyte is the most-used in vitro model for adipogenesis and lipogenesis study. Lipogenesis takes place simultaneously with adipogenesis during the adipocyte differentiation, which is controlled by sequential expression of PPARγ, C/EBPα, β and SREBPs, the master regulator of transcription factors for adipogenesis [1]. In addition, mitochondria transporters such as uncoupling proteins (UCP) are also known involved. UCP2 and UCP3 are linked in pathological conditions in obesity, diabetes and atherosclerosis [2,3]. Recently, mitochondrial solute carrier family genes were reported of playing a role during adipogenesis [4-7], but their role in lipid metabolism is unclear and still under investigation. Understanding the functional roles of gene regulated during adipocyte differentiation becomes a major challenge in the post-genome era. Although many genes that participate in fat metabolism are already discovered, the existence of yet-to-be identified genes important for adipogenesis is quite possible, as, to our knowledge, no systematic functional screening has been done for adipogenic genes. Genome-wide gene expression analysis and profiling using gene-trapping, RNA interference, and microarray analysis to identify expressed genes for adipogenesis have been reported [8-11]. However, these approaches were unable to link a phenotype change to the expression of a specific gene in a given cell. In the present study, we took a novel systematic approach to identify functional genes in adipocyte differentiation at whole-genome scale, based on the principle of random homologous knockdown (RHKD) [12] by using lentivirus-based tet-inducible vector system for mRNA knockdown. Anti-sense RNAs generated upstream of randomly inserted lentivector through a reverse oriented promoter in each cell's genome will disrupt the synthesis of protein from mRNA generated upstream of lentivector insertion site (of both alleles) by interference with the translation or stability of mRNA. By monitoring the loss of a function or a phenotype (e.g. failure to differentiate) of individual cells after RHKD intervention, the gene implicated in the function or phenotype change can be identified, cloned and characterized.

Mitochondrial oxodicarboxylate carrier (ODC) was first identified and characterized by Fiermonte et al. in Saccharomyces cerevisiae [13] and then in human and rat, which catalyzes the transport of 2-oxoadipate and 2-oxoglutarate by a counter-exchange mechanism [14]. In the present study, an alternatively-spliced form of murine ODC (ODC-AS) was identified in 3T3-L1 cells through the RHKD approach [GenBank accession number EU180236]. The expression of ODC-AS and ODC in mouse tissues was investigated and its role in adipogenesis in 3T3-L1 cells and in fat metabolism in mice was confirmed.

## Methods

### Cell culture and differentiation

We used the well established 3T3-L1 cell line as a model system for adipocyte differentiation. 3T3-L1 preadipocytes (ATCC, Manassas, VA) were cultured in Dulbecco's modified Eagle's medium (DMEM, Invitrogen, Carlsbad, CA) containing 10% fetal bovine serum (FBS, Invitrogen). Cell differentiation into adipocytes was induced at confluence 2 days after, with DMEM containing 10 μg/ml insulin (Invitrogen), 0.5 mM 3-isobutyl-1-methylxantine (IBMX, Sigma-Aldrich, St. Louis, MO) and 1 μM dexamethasone (DEX, Sigma-Aldrich) for 2 days, and then with DMEM supplemented with 10% FBS and 1 μg/ml insulin only for another 2 days. After induction, the cells were fed every other day with DMEM containing 10% FBS. Differentiation was verified by accumulation of lipids within the cells as detected by Nile Red (Sigma-Aldrich) staining.

### Functional gene screening during 3T3-L1 cell differentiation

**Figure 1:**
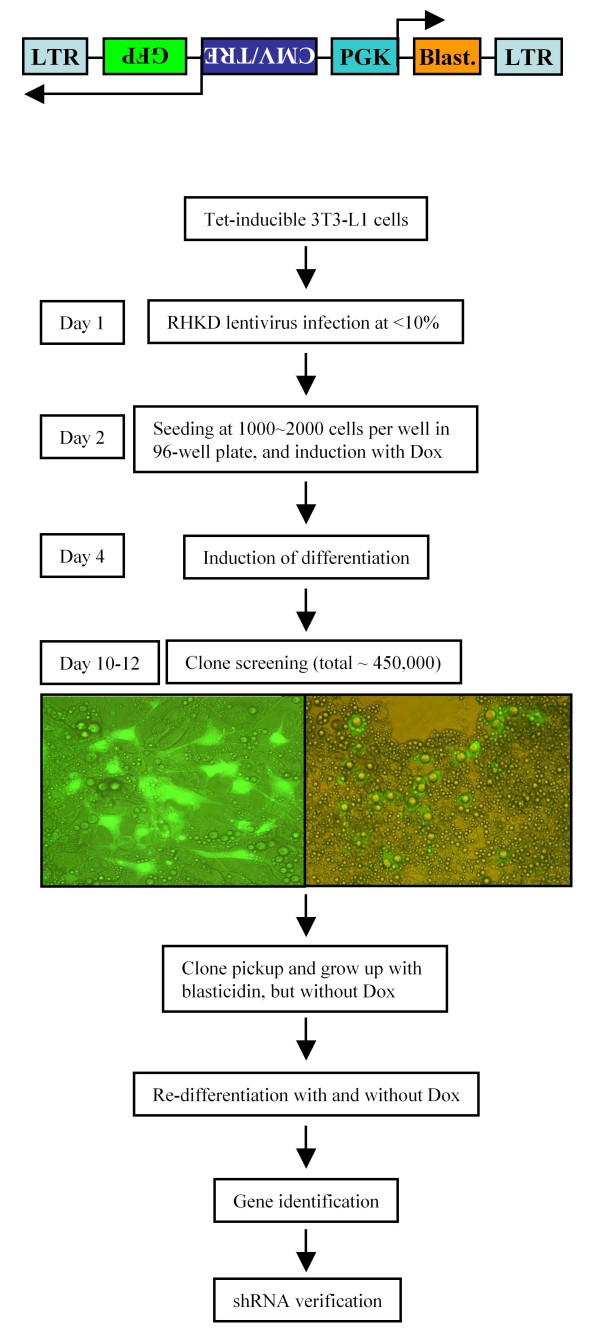
**Procedures of clone screening after RHKD**. RHKD tet-inducible lentiviral vector design (top) and screening procedure of 3T3-L1 cells for adipogenesis with RHKD. CMV/TRE, cytomegalovirus/tetracycline response element; GFP, green fluorescent protein; PGK, phosphoglycerate kinase promoter; Blast, blasticidin-S deaminase for selection against blasticidin after lentiviral infection; LTR, long terminal repeat. Left microphotograph shows a clone of 3T3-L1 cells (GFP-positive) after RHKD, which have little lipid accumulation inside the cells, surrounded by non-infected (GFP-negative) and properly differentiated 3T3-L1 adipocytes. Right microphotograph shows a clone of 3T3-L1 cells (GFP-positive) after RHKD, which have over accumulated lipid inside the cells, compared to surrounding non-infected (GFP-negative) and properly differentiated 3T3-L1 adipocytes.

1) Tet-on transcriptional activator (TA) 3T3-L1 cell line establishment: 3T3-L1 cells were infected with lentiviruses containing tet-on TA for 24 hr in Opti-MEM (Invitrogen) in a Biosafety level-3 laboratory. After washing with normal culture medium and limiting dilution in 96-well plate, puromycin (1.25 μg/ml, Invitrogen) was added into culture medium for four days to select drug resistant clones. After expanding, tet-on 3T3-L1 clones were further infected with lentiviruses containing inducible luciferase and evaluated. Finally individual clones with minimum leakage (low basal luciferase levels without induction) and maximal induction were selected by measuring luciferase activity in the presence of 1 μg/ml doxycycline (Dox, Sigma-Aldrich).

2) Construction of lentiviral tet-inducible RHKD vector: CMV-tet response element (TRE) promoter was positioned in a reverse orientation close to the 5' LTR of provirus, according to the principle as described previously [12]. Green fluorescent protein (GFP) gene as a reporter was inserted downstream to the CMV-tet response element in a reverse orientation as well, so when the inducible promoter was activated, GFP was expressed and the infected cells can be identified with green fluorescence under fluorescent microscope (Fig. 1 top). Because there is no polyA(+) signal after GFP, the synthesis of GFP mRNA will continue reversely into the genomic DNA of hosting cells where the lentivirus was inserted. In such a way, anti-sense RNA was synthesized which is complementary to the mRNA derived from the sense strand of genomic DNA template adjacent to the 5'LTR of the inserted lentivirus.

3) Functional screening: Following a protocol schematically outlined in Fig. 1, Tet-on 3T3-L1 cells were infected with RHKD lentivirus at an infection rate of <10% in order to obtain single copy integration. Next day the cells were seeded at 1,000~2,000 cells in 100 μl medium per well in a 96-well plate in normal culture medium plus Dox (1 μg/ml). Two days after at confluence, cell differentiation was induced in the presence of Dox. Screening and clone selection were performed day 6 ~day 8 after differentiation. By observing cells under fluorescence microscope together with dim transmitted light, the degree of cell differentiation (lipid accumulation) in GFP expressing cells was compared with surrounding cells. GFP^+ ^clones (usually a group of 10~30 cells) with significant altered lipid accumulation were isolated by focal applied trypsin with a small piece of trypsin-pre-soaked filter paper (0.2 mm in diameter). After cell population grew up in the presence of blasticidin (12.5 μg/mL, Invitrogen), each clone was induced for re-differentiation in the absence and presence of Dox to confirm lipid formation (either with reduced or enhanced lipid accumulation in the presence of Dox, but normal in the absence of Dox) with Nile Red staining, and selected for gene (interfered) identification.

4) Modified 3'RACE and identification of lentivirus integration site in genome: Total RNA was extracted from the selected clones, using QuickPrep micro mRNA purification kit (Amersham, Piscataway, NJ). Reverse transcription was performed by using SuperScript II reverse transcriptase (Invitrogen) and a designed random-adaptor (5'-TGC AAC GAT ACG CTA CGT AAC GAG CTG ACA GTG NNN NN-3'). The derived cDNAs were used as template for nested PCR (5' vector-outer: 5'-GAG AGA GCT CCT CTG GTT TC-3' with 3'outer-adaptor: 5'-CAA CGA TAC GCT ACG TAA CG-3'; and next round 5' vector-inner: 5'-TCG CTT TCA AGT CCC TGT TC-3' plus 3'inner-adaptor: 5'-TAC GTA ACG AGC TGA CAG TG-3') to amplify the junction region of the genomic DNA with the 5'LTR of the inserted lentivirus. Finally the amplified PCR products were cloned into pCR4-TOPO vector (Invitrogen) and sequenced using M13 reverse and/or M13 forward primers.

### Cloning of ODC-AS cDNA

Cloning of the full coding region of ODC from 3T3-L1 cells with conventional PCR and primers based on GenBank sequence (NM_172577) was not successful, we then used 5' rapid amplification of cDNA end (RACE) to obtain the 5'-end of the ODC transcript. One reverse primer (5'-ATCGCTTCTCAGTCCATCAC-3') was used for the synthesis of cDNA for 5'RACE, with total RNA extracted from differentiated 3T3-L1 cells. The 5' end fragment of ODC was amplified with nested PCR (GeneRace Core kit, Invitrogen, 5'-outer and 5'-inner from kit plus 3'-ODC-outer: 5'-CGATTGACTTGCAAGCCAAC-3' and 3'-ODC-inner: 5'-GAAAGGGTTAACCACGACAG-3') and cloned into pCR4-TOPO vector and fully sequenced. Finally the full length cDNA of ODC-AS was cloned by RT-PCR using gene-specific primers.

### Multiple-tissue ODC and ODC-AS expression profiling

Expression of ODC and ODC-AS was analyzed by PCR amplification of mouse Multiple Tissue cDNA panel (MTC, Clontech, Palo Alto, CA) with specific primers (5'ODC: 5'-CCCTGGTCGCGAAGATGTC-3', 5'ODC-AS: 5'-CAGTCGACCTACGTACCATG-3' and common 3' primer: 5'-CGATTGACTTGCAAGCCAAC-3') at the following conditions: 40 cycles of 95°C for 30 seconds, 68°C for 90 seconds with extension at 72°C for 3 minutes using Advantage II PCR kit (Clontech) (Additional file 1). The amplified products at the expected size of 457 bp for ODC-AS and 433 bp for ODC respectively, were separated on 1% agarose gel with ethidium bromide and visualized under UV excitation. Additionally, eye balls from 10 adult mice were collected postmordem and micro-dissected into six portions on ice: cornea, iris, lens, retina, sclera-choroid complex and optic nerve. Total RNA was extracted from the respective tissues and used for the detection of ODC and ODC-AS expression with RT-PCR.

### Silence of ODC-AS with RNA interference and real-time RT-PCR(qRT-PCR)

The ODC/ODC-AS specific short hairpin oligonucleotides were 19 bases long with TTCAAGAGA as loop sequence, designed using computer software. Total inserts about 50 bases long of both strands were synthesized by Integrated DNA Technology (Coralville, IA). After annealing, the double strand inserts were directly ligated into lentivector in which the synthesis of the shRNA is driven by a human U6 promoter. The knockdown efficiency of 6 shRNA constructs was evaluated first against ODC-AS-GFP fusion protein expression in stable HEK-293 cells, based on green fluorescence levels 5 days after shRNA introduction. Three shRNAs against ODC-AS with nucleotide sequences (#1: 5'-GAGGCAGCTTCCAGATGAT-3' with 42% knockdown efficiency; #3: 5'-GCAGCTTCCAGATGATCTT-3' with 69% knockdown efficiency and #5: 5'-GGAGAGATCAAGTACCGAAG-3' with 96% knockdown efficiency) were chosen. Three days after lentivirus infection with the selected shRNA, the 3T3-L1 cells were induced for differentiation. The shRNA against PPARγ (5'-GACATTCCATTCACAAGAG-3') was used as positive control and a non-specific shRNA (Nsi, 5'-TCAGTCACGTTAATGGTCG-3') which is known not to match any known genes was used as negative control. Total RNA was extracted from homogenized 3T3-L1 cells using the Tri reagent (T9424, Sigma-Aldrich), according to the manufacturer's instruction. qRT-PCR was conducted using ABI 7900 real-time PCR system (Applied Biosystems, Foster City, CA). The Taqman primers and probes were ordered from the Applied Biosystem: PPARγ (Mm00440945_m1), aP2 (Mm00445890_m1), CD36 (Mm00432403_m1). C/EBPα mRNA was detected using iTaq™ SYBR^® ^Green kit from BIO-RAD (Hercules, CA). The primers for C/EBPα are: forward, 5'-GCGAGCACGAGACGTCTATAGA-3'; reverse, 5'- GCCAGGAACTCGTCGTTGAA-3'. A mean value of triplicates was used to represent relative mRNA level or calculate mRNA fold induction.

### Triacyl glycerol (TAG) quantification in adipocytes

Adipocytes (6 days after differentiation of 3T3-L1 cells) were harvested in TNET buffer (50 mM Tris-HCl, pH 7.4, 150 mM NaCl, 2 mM EDTA, and 1% Triton X-100) plus 0.5% deoxycholate. TAG was measured using Serum Triglyceride Determination Kit (Sigma-Aldrich) by following manufacturer's instruction. To normalize TAG for protein, the protein content was quantified using the DC-Protein Assay Reagents (BIO-RAD, Hercules, CA).

### Cold exposure and qRT-PCR of ODC and ODC-AS

Two month old female C57BL/6 mice, n = 5 each group, were kept either at room temperature or at 4°C for 18 hours, as approved by institutional IACUC committee. Then white (ovarian) and brown adipose tissues were collected, and the expression of ODC and ODC-AS was quantified by real-time PCR, using iTaq™ SYBR^® ^Green kit (BIO-RAD). The mRNA signal was normalized over G3PDH signal. A mean value of triplicates was used to represent relative mRNA level or calculate mRNA fold increase.

## Results

### Functional gene screening for adipogenesis with RHKD

Using the 96-well-based screening approach, over estimated 300,000 3T3-L1 cells or clones from 30 96-well plates (10% of seeded 1,000-2,000 cells per well) after RHKD were screened. Total seven clones with altered lipid accumulation (one with enhanced lipid accumulation and six with no or reduced lipid formation) after differentiation were identified. Two clones failed to grow up after trypsinization and isolation, and one clone could not re-differentiate after growing up, even in the absence of Dox. The remaining four clones were subject to gene identification after re-differentiation confirmation, by cloning the cDNA fragment downstream of GFP with modified 3'RACE. All four clones have single lentivirus integration into the genome, located in chromosome 9, 11, 12 and 18, and either in the middle of a gene (in an intron) or within 100 kb downstream of an open reading frame (ORF), respectively. Among them, clone LL4 was found with reduced lipid formation, with a single lentivector integrated between exon 8 and 9 of ODC gene [GenBank Accession #: NM_172577]. Since the direction and initiation site of the generated anti-sense RNA was correct, we reasoned that ODC was homologously knocked down in LL4 cells, which caused the phenotype change during adipogenesis.

### Identification, cloning and expression of ODC-AS

To isolate the full length cDNA of ODC-AS from LL4 clone, we performed 5'RACE with primers designed based on GenBank sequence for ODC. Full-length cloning of the ODC-AS transcripts revealed that the cDNA fragment is 918 bp long and encodes a 305 aa peptide with the 29 aa replacing 22 aa in mouse ODC at N terminal. The cDNA for ODC-AS includes 69 bp 5'-untranslated region, with an in-frame stop codon preceding the ATG start codon. Genomic analysis confirmed that ODC-AS derived from two additional exons (2 and 3), and both ODC (exon 1 only) and ODC-AS share the common exon 4-12 (Additional file 1). Hence, the 3T3-L1 variant of ODC transcript is resulted from alternate splicing (we thus named this ODC transcript as ODC-AS). Using both PSORT II program and NNPSL algorithm (Sanger Centre, Hinxton, Cambridge, UK), ODC-AS, as ODC was shown previously [14], is also predicted to locate in the mitochondrial membrane.

**Figure 2:**
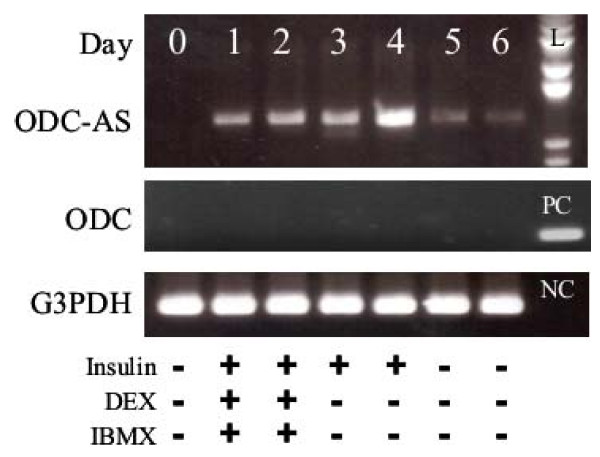
**RT-PCR analysis of ODC-AS expression in 3T3-L1 cells**. Expression of ODC-AS and ODC mRNA in 3T3-L1 Cells were detected with RT-PCR in mRNAs prepared at the indicated days from 3T3-L1 cell pre- and after differentiation, and visualized under UV after agarose gel electrophoresis with ethidium bromide. 1 kb DNA standards is shown on the right. The addition of differentiation reagents was indicated at the bottom. G3PDH was amplified as an internal control and shown below. L: 1 kb DNA ladder; PC: positive control for ODC from cloned cDNA plasmid; NC: negative control for G3PDH (water).

To examine the expression, RT-PCR was performed with cDNA from 3T3-L1 cells collected at pre- and during differentiation (day 1 to day 6). ODC expression was not detected in pre-, differentiating and differentiated 3T3-L1 adipocytes from three separate experiments. In the same samples, ODC-AS gene was expressed from day 1 after differentiation induction and reached a maximal level at day 4 (Fig. 2). Lower level of expression was still seen 10 days after differentiation induction from additional experiments. The pattern of expression strongly suggests that ODC-AS may be involved in the regulation of adipogenesis. Next, we examined the expression of ODC-AS gene by RT-PCR in various murine tissues (heart, brain, spleen, lung, liver, skeletal muscle, kidney, testis, ES cells, 7 day embryo, 11 day embryo, 15 day embryo, 17 day embryo, bone marrow, eye, lymph node, smooth muscle, prostate, thymus, stomach, uterus) using MTC from Clontech. ODC was detected in all mouse tissues studied, which is in agreement with a previous study [14]. However, ODC-AS expression was only observed in embryo at day 7 and eye tissue (Additional file 2). Subsequent experiments showed that both ODC and ODC-AS were expressed in white and brown adipose tissues. In the eye (dissected into cornea, iris, lens, retina, sclera-choroid complex and optic nerve for cDNA preparation), RT-PCR results showed that ODC-AS was only expressed in iris and sclera-choroid complex, whereas ODC was expressed in all 6 separated eye tissues (Additional file 3).

### Knock down of ODC-AS impairs adipogenesis

**Figure 3:**
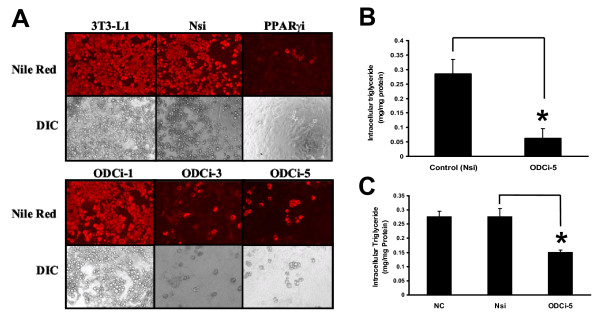
**Knockdown of ODC-AS by RNA interference**. A. Phenotype changes in adipogenesis by shRNA knockdown of ODC-AS. Microphotographs of 3T3-L1 cells infected with shRNA (ODCi-1, ODCi-3 and ODCi-5) for 3 days before differentiation cocktail was added, and the cells were stained with Nile Red 6 days after differentiation. Nsi. Non-specific shRNA served as a negative control; PPARγi is a shRNA designed specifically against PPARγ, served as a positive control to inhibit 3T3-L1 differentiation. B. Quantification of intracellular triglyceride in ODCi-5-infected 3T3-L1 and Nsi control cells. Cells were infected 3 days before differentiation and samples were collected 6 days after differentiation. Data shown are means ± S.D. and were in triplicates of three separate experiments. C. Quantification of intracellular triglyceride in ODCi-5-infected, Nsi-infected 3T3-L1cells and normal control (NC, non-infected). The cells were infected 2 days after the induction of differentiation and samples were collected 10 days after differentiation. Data shown are means ± S.D. and were in triplicates of three separate experiments. * indicates P < 0.01, Student *t *test.

**Figure 4:**
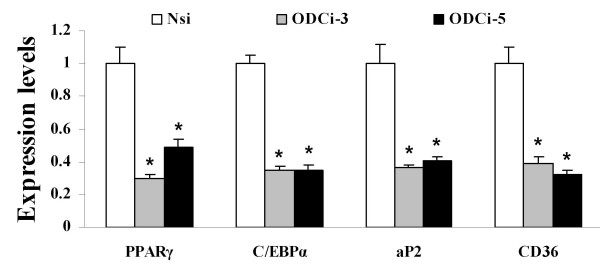
**ODC-AS knockdown and qRT-PCR of marker genes in adipogenesis**. 3T3-L1 preadipocytes were infected with lentivirus (Nsi, ODCi-3 or ODCi-5) before the induction for differentiation. Twenty four hours after induction, the cells were collected and examined for mRNA levels of PPARγ, C/EBPα, aP2 and CD36 (see Methods). Data were expressed as means ± SEM (n = 3), relative to Nsi. *compared with Nsi: *P *< 0.001.

To determine the role of ODC-AS in adipogenesis, we designed 6 different shRNAs and selected 3 (ODCi-1, ODCi-3 and ODCi-5) to knockdown ODC-AS expression in 3T3-L1 cells, together with Nsi as a negative control and a shRNA against PPARγ as positive control. These shRNAs were introduced into 3T3-L1 cells by lentiviral infection 3 days before the induction of adipogenesis. As shown in Fig 3A, ODCi-1 which showed weak reduction of fusion ODC-AS-GFP expression did not show clear inhibitory effect on adipogenesis with Nile Red staining. However, ODCi-3 and ODCi-5 significant inhibited the cell differentiation. The cells had much less lipid accumulated inside the cells shown with Nile Red staining 6 days after the induction of differentiation. Direct measurement of intracellular triglyceride content showed that lipid level was reduced by 78.4% with ODC-AS knockdown (0.06 ± 0.03 mg/mg protein for ODCi-5 with comparison to 0.29 ± 0.05 mg/mg protein of Nsi control. n = 3, P < 0.01, Student ***t ***test) (Fig. 3B). Similar results were obtained when 3T3-L1 cells were infected with ODCi-5 lentivirus 2 days after the cell differentiation was induced (Fig. 3C). In this case, highly concentrated lentiviruses were used in order to have majority of the cells infected. Intracellular triglyceride was measured on day 10. To further confirm the function of ODC-AS in adipogenic gene expression during adipocyte differentiation, 3T3-L1 preadipocytes were infected with lentivirus (Nsi, ODCi-3 or ODCi-5) for 24 hours before induction of differentiation into mature adipocytes. mRNA levels of adipogenic marker genes including PPARγ, aP2, CD36 and C/EBPα were determined using qRT-PCR. The results showed that both ODCi-3 and ODCi-5 inhibited the expression of these adipogenic genes significantly. PPARγ was reduced by 70.4% and 51.2% with ODCi-3 and ODCi-5, respectively (*P *< 0.001). Gene expression of C/EBPα, aP2 and CD36 were all reduced by more than 60% with ODC-AS knockdown (*P *< 0.001). The data by knocking down of ODC-AS indicate that inhibition of adipogenesis may be through the suppression of adipogenic genes (Fig. 4).

**Figure 5:**
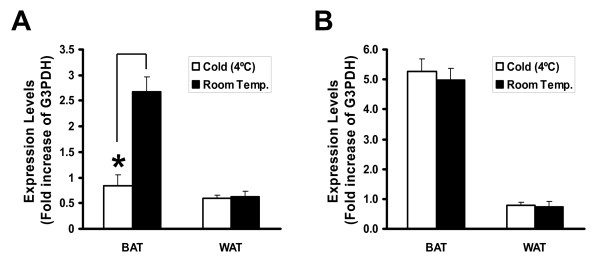
**ODC and ODC-AS expression in fat tissues**. A. ODC-AS gene expression in fat tissues by cold exposure. ODC-AS expression in brown adipose tissue (BAT) and white adipose tissue (WAT) as quantified by real-time PCR and expressed as the fold increase of G3PDH, after 18 hour exposure of mice at 4°C and room temperature. Data shown are means ± S.D, n = 5. * indicates P < 0.01, Student *t *test. B. ODC expression in BAT and WAT as quantified by real-time PCR and expressed as the fold increase of G3PDH, after 18 hour exposure of mice at 4°C and room temperature. Data shown are means ± S.D, n = 5.

To test whether expression levels of ODC-AS are subject to regulation by cold exposure, we compared ODC-AS gene expression in female mice (C57BL/6, n = 5 each group) which have been exposed to 4°C for 18 hours. Compared to the room temperature controls, ODC-AS expression in brown adipose tissue (BAT) was significantly reduced upon cold exposure as determined by real-time PCR (average 0.84 folds of G3PDH vs. average 2.68 folds at room temperature, P < 0.01, Student ***t ***test), whereas no changes of expression levels were observed in white adipose tissue (WAT) (Fig. 5A). No change in the expression level of ODC was observed after cold exposure, neither in BAT nor in WAT (Fig. 5B).

## Discussion

In the present study, we have demonstrated that RHKD can be used to discover new functional genes for adipogenesis using 3T3-L1 cells. Further, we have identified a novel cDNA transcript, ODC-AS, which is an alternatively spliced form of ODC gene [14]) from our functional genomic analysis during 3T3-L1 differentiation. Further more, we showed that ODC-AS is only expressed in adult eye and adipose tissues. The biological reasons for the co-existence of these two alternatively spliced forms in eye and fat tissue are not clear. In analogy, the mitochondrial phosphate carrier (PiC) (SLC25A3) also has two alternatively spliced variants, different in the N-terminus region [15]. PiC-A showed tissue specific expression and PiC-B showed ubiquitous expression. In addition, these two isoforms of PiC exhibited different transport affinity and activity. The difference in expression and activity of PiC variants was suggested to be responsible for the energy demand in the different tissues [16]. At the pre-adipocyte stage of 3T3-L1 cells, neither ODC nor ODC-AS are expressed in the cells. Only ODC-AS expression was observed during and after cell differentiation (Fig. 2). Functional characterization of ODC has already been reported previously [14,17-21]. ODC is shown to catalyze the uptake of 2-oxoadipate and 2-oxoglutarate into the mitochondria by a counter exchange mechanism. In addition, adipate, glutarate, and pimelate, 2-oxopimelate, 2-oxoaminoadipate, oxaloacetate and citrate are transported through ODC. Since 2-oxoadipate is an intermediate in the catabolism of lysine, tryptophan and hydroxylysine and ODC is expressed in all the tissues, ODC is considered to be essential for amino acid metabolism [14]. However, there is no reported role for ODC related to adipogenesis or lipogenesis. In 3T3-L1 cells which do not express ODC, the determination of transport activity of ODC-AS would help to understand how ODC-AS influences adipogenesis. By using RNA interference to knockdown ODC-AS gene expression in the 3T3-L1 cells, cell differentiation and lipid accumulation was inhibited. Our data on the adipgenic gene expression levels for PPARγ, aP2, CD36 and C/EBPα after ODC-AS knockdown suggest that ODC-AS may act on adipogenesis. The knockdown of ODC-AS may limit the transport of oxaloacetate out of the mitochondria, thus limit glyceroneogenesis and further the formation of glycerol-3-phosphate backbone for triacylglycerol formation. The knockdown of ODC-AS may also limit the transport of citrate, which is required for fatty acid synthesis. Further metabolic experiments will help delineate between these potential mechanisms. We attempted to infect the cells with shRNA 6 days after the induction of differentiation in order to determine specifically if ODC-AS plays a role in lipogenesis (to differentiate from adipogenesis), but only a few cells could be infected, even using highly concentrated lentivirus stock. To further confirm the function of ODC-AS in adipogenic gene expression during adipocyte differentiation, the effect on adipogenic marker genes such as PPARγ, aP2, CD36 and C/EBPα was also determined by real-time RT-PCR and further confirmed the role of ODC-AS in adipocyte differentiation.

Genes like UCP1 participated in fat oxidation and mobilization were induced for thermogenesis in brown fat when animals were exposed to cold [22]. ODC-AS gene expression was down-regulated by cold exposure in the present study, suggesting it may be involved in fat utilization. Since both ODC and ODC-AS are expressed in the BAT but only ODC-AS expression was affected by cold, it strongly suggests that the functional role and substrate preference for transport by ODC-AS is different from ODC, especially for the unique tissue expression in adipose tissue and eye. However, it is well known that C/EBPα and PPARγ play critical role in adipocyte differentiation in both white and brown fat tissues. ODC-AS knockdown reduced the expression of both in white adipocytes. We expect the same effect in the brown adipocyte differentiation. Because the difficulty to obtain antibodies recognizing ODC and ODC-AS specifically, no information regarding the tissue and cellular locations of ODC and ODC-AS could be determined. The expression of ODC-AS-GFP in 3T3-L1 cells, as judged by the presence of green fluorescence, seems confined to cytosolic organells (possibly mitochondria), consistent with computer-assisted prediction (data not shown).

ODC belongs to the SLC25 gene family of mitochondrial transporter family [17]. Recently, Slc25a10 (DIC) and Slc25a11 (OGC) have been reported to participate in adipogenesis, lipid accumulation and fatty acid synthesis [4-7]. Mizuarai et al showed that DIC, a malate/citrate transporter, play an important role for fatty acid synthesis. The expression of DIC was also induced when 3T3-L1 cells differentiated into adipocytes, and the suppression of its expression down-regulated fatty acid synthesis and reduced lipid accumulation, which may be due to reduced supply of malate for the citrate transport required for fatty acid synthesis [4]. It is interesting to note that DIC was up-regulated in three obese mouse models and down-regulated in the lean mouse model. DIC is expressed at both murine BAT and WAT with very high levels in mitochondria of WAT. In addition, exposure to cold down-regulated DIC levels in fat tissues *in vivo *[7]. In another study, OGC, a 2-oxoglutarate carrier, was shown up-regulated in BAT of mice exposed to cold for 48 hours [6]. Although the functional aspect of ODC-AS as a mitochondrial carrier is unclear, the results presented in the present study indicate that ODC-AS may play a similar role in adipocyte differentiation and lipid synthesis and utilization. Our gene silence data indicate that ODC-AS and adipogenic genes are positively correlated. Taking into the consideration of the role of brown fat in energy generation, the reduction of ODC-AS expression may be due to reduced alternative splicing, through feedback mechanism, though we do not understand how the alternative splicing is regulated.

Over the years, many laboratories have used 3T3-L1 cells for the study of adipogenesis and lipogenesis, as the 3T3-L1 pre-adipocyte cell line is the best characterized. Although ODC has been found expressed ubiquitously in all the tissues studied including adipose tissue [[14] and the present study], we were surprised by detecting no ODC expression in these 3T3-L1 cells. We tried to assess the function of ODC in adipogenesis by over-expressing ODC in the cells and established stable cell lines, in order to answer the question whether the lack of ODC expression in these cells make them adipogenic. However, neither inhibition nor enhancement of cell differentiation after induction for adipogenesis was observed with the constitutive ODC-expressing cells, compared to non-infected normal controls (data not shown). Further, no obvious effect on 3T3-L1 adipogenesis was observed by heterogenous over-expression of ODC-AS, suggesting that the endogenous level of its expression after differentiation induction is sufficient for adipogenesis or lipogenesis.

Identification of new genes in the adipogenesis may help to identify novel anti-obesity targets, as suppression of the expression of acetyl-CoA synthetase with RNA interference was shown with reduced fat levels in *Caenorhabditis elegans *[23]. Reduced lipid accumulation by knocking down ODC-AS through RNA interference indicated that ODC-AS could be a potential target for anti-obesity intervention, especially for its unique expression in adipose tissue and eye. It is interesting to point out that both iris and sclera-choroid complex share a common cell type called pigment epithelium, which are very important for photoreceptor function and are the pathological site of age-related macular degeneration [24].

## Conclusion

Through the functional genomic search using RHKD approach, we identified an alternatively spliced form of ODC which is expressed after 3T3-L1 cell differentiation and in adipose tissues. ODC-AS expression is required for adipogenesis in 3T3-L1 adipocytes. Further investigation is warranted to elucidate its biochemical and biological role and its relationship to obesity and diabetes-related complications.

## Competing interests

The authors declare that they have no competing interests.

## Authors' contributions

MN, LT, SL and JY performed gene screening, cloning and characterization; XW designed and constructed RHKD vectors; HF, JK, JY and BS designed the experiments, performed data analysis and wrote the manuscript.

## Supplementary Material

Additional file 1 **Genomic structure and alternative splicing of ODC gene**. Schematic diagram of mouse genomic structure and splicing difference between ODC and ODC-AS in the 5' region was shown. The specific forward primers used specifically for ODC and ODC-AS are indicated respectively, as well as the common reverse primer. Click here for file

Additional file 2 **Expression of ODC and ODC-AS in murine tissues**. PCR amplification of ODC and ODC-AS from cDNAs of murine multiple-tissue panel (Clontech) with primers as shown in Additional file 1, was visualized under UV after being separated on 1% agarose gel containing ethidium bromide. The expected size of the bands was shown on the left and the DNA 1 kb ladder was shown on the right. Click here for file

Additional file 3 **Expression of ODC and ODC-AS in the mouse eye**. PCR amplification of ODC and ODC-AS from cDNAs of 6 dissected tissues from mouse eye with primers as shown in Additional file 1, was visualized under UV after being separated on 1% agarose gel containing ethidium bromide. The expected size of the bands was shown on the left and the DNA 1 kb ladder was shown on the right. Click here for file
