# Supplementary figures and images for: Involvement of an alternatively spliced mitochondrial oxodicarboxylate carrier in adipogenesis in 3T3-L1 cells
Source: J Biomed Sci. 2009 Oct 13;16(1):92. doi: 10.1186/1423-0127-16-92 (PMC2765418; doi:10.1186/1423-0127-16-92)

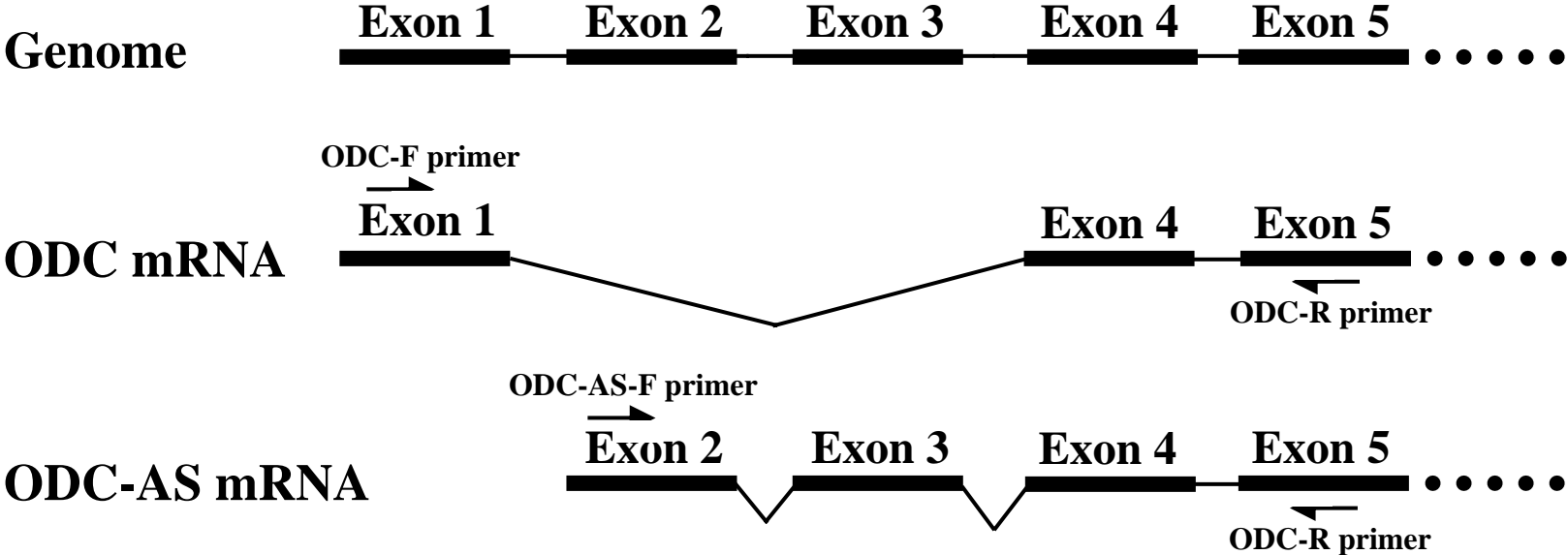

Supplement: Additional file 1 — Genomic structure and alternative splicing of ODC gene. Schematic diagram of mouse genomic structure and splicing difference between ODC and ODC-AS in the 5' region was shown. The specific forward primers used specifically for ODC and ODC-AS are indicated respectively, as well as the common reverse primer. [file 1423-0127-16-92-S1.PDF]
