# Supplementary material for: Involvement of an alternatively spliced mitochondrial oxodicarboxylate carrier in adipogenesis in 3T3-L1 cells
Source: J Biomed Sci. 2009 Oct 13;16(1):92. doi: 10.1186/1423-0127-16-92 (PMC2765418; doi:10.1186/1423-0127-16-92)
Supplement: Additional file 2 — Expression of ODC and ODC-AS in murine tissues. PCR amplification of ODC and ODC-AS from cDNAs of murine multiple-tissue panel (Clontech) with primers as shown in Additional file 1, was visualized under UV after being separated on 1% agarose gel containing ethidium bromide. The expected size of the bands was shown on the left and the DNA 1 kb ladder was shown on the right. [file 1423-0127-16-92-S2.PDF]

M. Niimi et al. Supplement 2: Expression of ODC and ODC-AS in murine tissues

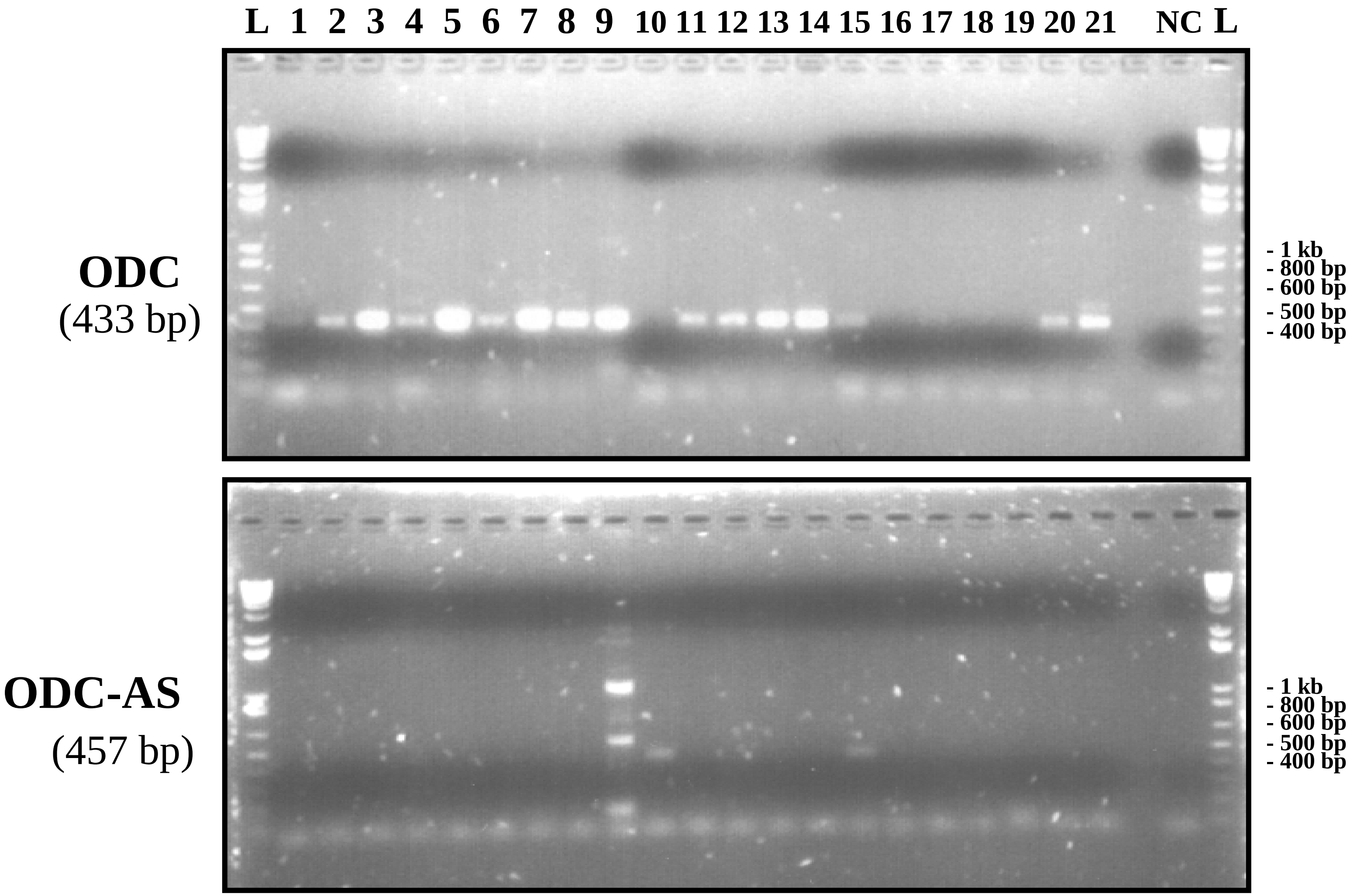

1. Heart; 2. Brain; 3. Spleen; 4. Lung; 5. Liver; 6. Skeletal muscle; 7. Kidney; 8. Testis; 9. ES cells; 10. 7day embryo; 11. 11day embryo; 12. 15day embryo; 13. 17 day embryo; 14. Bone marrow; 15. Eye; 16. Lymph node; 17. Smooth muscle; 18. Prostate; 19. Thymus; 20. Stomach; 21. Uterus; NC, negative control (water); L: 1kb DNA ladder
