# Supplementary material for: Involvement of an alternatively spliced mitochondrial oxodicarboxylate carrier in adipogenesis in 3T3-L1 cells
Source: J Biomed Sci. 2009 Oct 13;16(1):92. doi: 10.1186/1423-0127-16-92 (PMC2765418; doi:10.1186/1423-0127-16-92)
Supplement: Additional file 3 — Expression of ODC and ODC-AS in the mouse eye. PCR amplification of ODC and ODC-AS from cDNAs of 6 dissected tissues from mouse eye with primers as shown in Additional file 1, was visualized under UV after being separated on 1% agarose gel containing ethidium bromide. The expected size of the bands was shown on the left and the DNA 1 kb ladder was shown on the right. [file 1423-0127-16-92-S3.PDF]

M. Niimi et al. Supplement 3: Expression of ODC and ODC-AS in the mouse eye

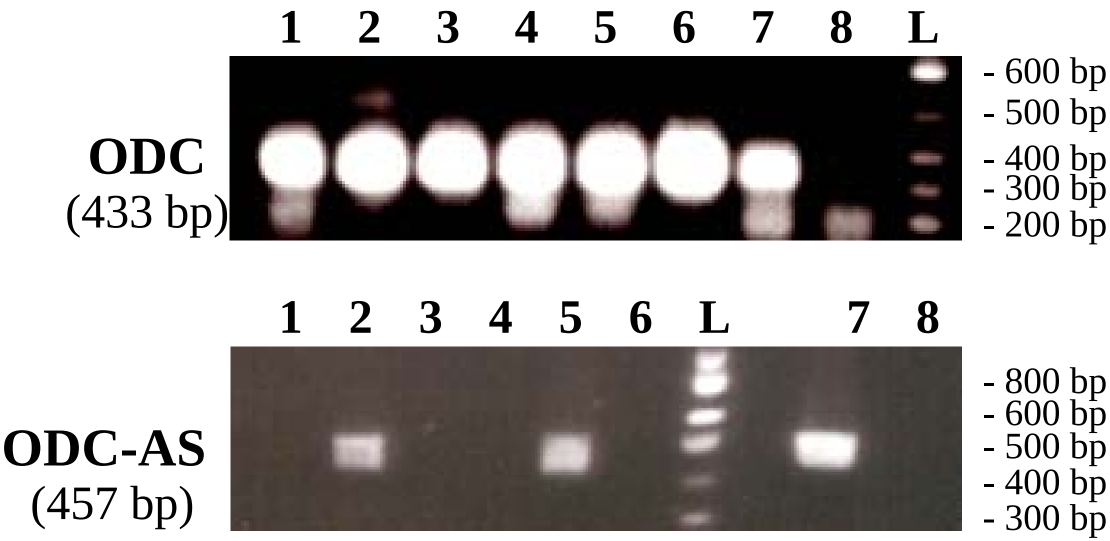

1. Cornea
2. Iris
3. Lens
4. Retina
5. Sclera-choroid complex
6. Optic nerve
7. Positive control (cloned cDNA)
8. Negative control (water)

L. 1kb DNA ladder
